# Can Antiretroviral Drugs Be Used to Treat Porcine Endogenous Retrovirus (PERV) Infection after Xenotransplantation?

**DOI:** 10.3390/v9080213

**Published:** 2017-08-08

**Authors:** Joachim Denner

**Affiliations:** Robert Koch Fellow, Robert Koch Institute, Nordufer 20, 13353 Berlin, Germany; DennerJ@rki.de; Tel.: +49-30-18754-2800

**Keywords:** porcine endogenous retroviruses, PERV, antiretroviral drugs, reverse transcriptase, integrase

## Abstract

Porcine endogenous retroviruses (PERVs) are integrated in the genome of all pigs; they are released as infectious particles, and under certain conditions they can infect human cells. Therefore, they represent a risk when pigs are used as sources of cells, tissues, or organs for xenotransplantation. Xenotransplantation is under development due to the increasing shortage of human transplants. Whereas most porcine microorganisms which may be able to induce a disease (zoonosis) in the transplant recipient can be eliminated, this is not possible in the case of PERVs. Antiretroviral drugs which had been developed for the treatment of human immunodeficiency virus-1 (HIV-1) infections have been tested in vitro for their efficacy in inhibiting PERV replication. Inhibitors of the viral reverse transcriptase and of the integrase have been found effective. The most effective inhibitor of the reverse transcriptase was azidothymidine (AZT); the integrase inhibitors were the most potent inhibitors of PERV. Although in the past PERV transmission has not been observed after experimental or clinical xenotransplantation of pig cells or organs, and although PERVs may one day be inactivated in pigs by genome editing using CRISPR/Cas, knowing which antiretroviral drugs can effectively restrict PERV infection will still be important.

## 1. Introduction

Xenotransplantation using pig cells, tissues, or organs is under development due to the permanent shortage of human organs for transplantation, and a broader clinical application of xenotransplantation products is expected in the near future [1,2,3]. Xenotransplantation may be associated with the transmission of porcine microorganisms able to induce zoonoses in the transplanted recipient [4,5]. Whereas most porcine bacteria, fungi, protozoa, and viruses may be eliminated by the selection and isolation of uninfected animals, treatment with antiviral drugs, vaccination, Cesarean delivery, early weaning, colostrum deprivation, and embryo transfer, this is impossible in the case of porcine endogenous retroviruses (PERVs). In contrast to exogenous retroviruses such as the human immunodeficiency virus (HIV), which infect only specific target cells and integrate their viral genome as an DNA copy called provirus into the genome of only these target cells, endogenous retroviruses are the result of a retroviral infection of oocytes and sperm cells, and therefore they are integrated in the genome of all cells of an organism [6]. There are three main types of PERVs integrated in the genome of pig cells: PERV-A and PERV-B are present in all pigs, and PERV-C is present in many pigs, but not all (for review see [7]). Whereas PERV-A and PERV-C can infect immortalized and in rare cases also primary human cells, PERV-C is an ecotropic virus infecting only pig cells [8,9,10,11]. Until now, no transmission of PERV has been observed in experimental xenotransplantation and PERV inoculations into small and non-human primates with and without immunosuppression (for review see [7]). PERV transmission was also not observed in the first clinical trials of porcine neonatal islet cell clusters for the treatment of diabetes performed in New Zealand and Argentina [12,13]. Despite this, different strategies to prevent PERV transmission during xenotransplantation or treat PERV infection have been initiated in the past. These strategies include PERV-specific vaccines [14,15,16,17], antiretroviral drugs [18,19,20,21,22,23,24,25], transgenic pigs expressing a PERV-specific small-interfering (si)RNA [26,27,28,29], and genome editing using zinc finger nucleases (ZFNs) [30] or CRISPR/Cas (clustered regularly interspaced short palindromic repeats, CRISPR-associated) [31]. Here a review on antiretroviral drugs acting efficiently against PERV will be given. These antiretroviral drugs were initially developed to inhibit other retroviruses (mainly HIV-1), and many have been licensed for the treatment of HIV infection. Although it may be possible to inactivate all proviruses by genome editing and to generate pigs not releasing infectious PERVs, it will still be useful to know which antiretroviral drugs are effective against PERV.

## 2. Life Cycle of PERV and Targets of Antiretroviral Drugs

**Figure 1:**
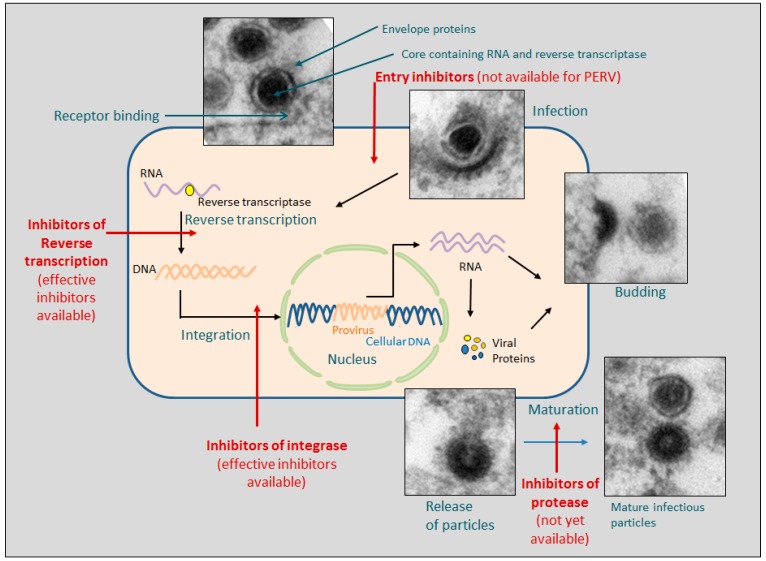
Life cycle of PERV and targets of four groups of antiretroviral drugs. It is indicated whether these inhibitors are available for treatment of PERV infection in vitro. The electron microscopic pictures show infection of human 293 cells by PERV and release of virus particles.

Retroviruses infect their target cells using one or two receptor(s), they transcribe their RNA genome into a DNA copy using the viral enzyme reverse transcriptase (RT), and integrate the DNA copy into the cellular genome using the viral enzyme integrase (Figure 1). Transcription of the proviral genes results in a full-length mRNA encoding for the Gag core proteins and the viral enzymes as well as a spliced mRNA encoding for the envelope (Env) proteins. Some retroviruses (e.g., HIV-1) have additional spliced mRNA encoding for accessory proteins. The translated proteins move to the cell surface, where the virus assembly takes place. After budding of the virus, the viral enzyme protease cleaves the Gag proteins, leading to the formation of the core and maturation of the virus. The life cycle of retroviruses including that of PERV can be interrupted at different steps acting on different viral targets in order to prevent virus replication [7] (Figure 1). First, viral entry can be blocked by substances of the drug class entry inhibitors. A subclass of these inhibitors interacts with the host co-receptor molecule (co-receptor antagonists) and prevents the binding of viral envelope protein. In the other subclass, the fusion inhibitors interact with viral structures to prevent the viral entry. Synthetic peptides binding to two helical domains in the transmembrane envelope protein hamper fusion with the cell membrane. Second, the RT which transcribes the retroviral RNA genome into a DNA copy is the target for two drug classes: (a) the nucleoside or nucleotide reverse transcriptase inhibitors (NRTI/NtRTI) and (b) the non-nucleoside reverse transcriptase inhibitors (NNRTIs) (for review see [32]) (Table 1). NRTIs are analogs of physiological deoxyribonucleosides competing as alternative substrate. The lack of a 3′-OH group on the deoxyribose sugar induces a chain-termination of DNA synthesis since phosphodiester bridges can no longer be built. NRTIs are pro-drugs, and must be metabolically converted by host-cell kinases to their corresponding active triphosphate derivates (NRTI-TPs). NtRTIs are similar to NRTIs, but are monophosphorylated, and therefore referred to as a nucleotide analog. The thymidine analogs azidothymidine (AZT, zidovudine) and stavudin (d4T), the cytidine analogs lamivudine (3TC) and emtricitabine (FTC), the adenosine analog didanosine (DDL) and the guanosine analog abacavir (ABC) were successfully used NRTIs to treat HIV-infections. NtRTI are tenofovir (TDF) and adefovir (TAF).

The third class, NNRTIs—also RT inhibitors—is chemically distinct from NRTIs, and unlike the NRTIs does not require intracellular metabolism for activity. Unlike NRTIs that do not directly inhibit RT, NNRTIs bind to a hydrophobic pocket in a subdomain of the enzyme and allosterically slow down DNA polymerization significantly. However, recent work has suggested that their inhibition of reverse transcription might also be due to effects on RT RNase H activity and/or triphosphate binding. First-generation NNRTIs were nevirapin (NVP) and efavirenz (EFV) introduced between 1996 and 1998, while etravirin (ETV, 2008), and rilpivirin (RPV, 2011) were developed later, therefore called second-generation NNRTI.

Substances interfering with the integrase belong to the fourth drug class. Integrase enables the integration of the proviral DNA copy into the host chromosomal DNA genome in at least four steps. First, binding of the integrase to viral DNA (pre-integration complex); second, 3′ processing of the dinucleotides at each end of the viral DNA; third, strand transfer from the cytoplasm through a nuclear pore into the cell’s nucleus and irreversible binding of viral and host chromosomal DNA; and fourth, gap repair. To date, all licensed integrase inhibitors raltegravir (RAL), dolutegravir (DTG) and elvitegravir (EVG) act as integrase strand transfer inhibitors (INSTIs).

Substances interfering with protease belong to a fifth drug class. Protease acts at a late stage of the life cycle, after budding of the newly produced virus from the cellular membrane, it cleaves the precursor Gag–Pol(polymerase)-polyprotein into subunits, allowing the maturation of the virus particles. If the protease is inhibited and proteolytic splicing is prevented, non-infectious virus particles will result (for review see [33]).

All groups of the here-described retroviral inhibitors have been successfully developed for the treatment of HIV-1-infections [34] and have been approved by the Food and Drug Administration (FDA) of the USA, the European Medicines Agency (EMEA), and respective agencies in Canada, Japan, and other countries. Applying only one substance usually results in the selection of resistance mutations in the viral genome. However, a combinatorial application of three substances out of two drug classes (combination antiretroviral therapy, cART, previously called highly active antiretroviral therapy, HAART) has been very effective in the treatment of HIV-1 with a life-long suppression of virus replication [33,34,35].

## 3. Inhibitors of Reverse Transcriptase

The first drug described to be effective against PERV was AZT [18,19]. 3TC and d4T—two other nucleoside analogues—did not effectively inhibit PERV [18] (Table 1). Already in 1974, AZT was reported by Ostertag et al. [36] to specifically target the Friend virus strain of murine leukemia virus—a virus closely related to PERV. AZT was also the first drug shown to be effective against HIV-1 [37], and it was approved by the FDA for the treatment of HIV infection in 1987 [38].

AZT is a thymidine analog, and it selectively inhibits the reverse transcriptase of all retroviruses. As described above, cellular enzymes convert AZT to its triphosphate, an active metabolite that inhibits DNA synthesis by the RT by chain termination. It also acts on cellular DNA polymerases, but inhibits HIV-reverse transcriptase much better than cellular DNA polymerases (for review see [39]).

The activity of AZT against PERV was later confirmed in three additional studies [20,22,25]. In the first of these studies, 11 antiretroviral drugs licensed for HIV-1 therapy were assessed for their activities against PERV [20]. AZT was the most effective drug; in all cases, the susceptibility of the PERV RT was lower when compared with the susceptibility of the HIV-1 RT. In the second study, AZT was also found to be the most active drug [22]. The order of potency was AZT, tenofovir, adefovir, and stavudine. In the third study, the NRTI AZT, and the NtRTI adefovir and tenofovir were shown to be effective against PERV [25] (Table 1).

The observed susceptibility of PERV to AZT is not surprising; as mentioned above, AZT has a broad range of activity against several retroviruses, and sequence analysis showed that the RT of the murine leukemia viruses and of the feline leukemia viruses—also susceptible to the treatment with AZT—share more than 70% homology to the RT of PERV [20]. HIV-1 and PERV share only 22.5% amino acid residues in the RT, however the sequence homology in the target domain of AZT is much higher.

Testing the susceptibility of a recombinant PERV RT produced in bacteria, to three NRTI including AZT and six NNRTI showed a susceptibility of PERV to AZT and two other NRTI, but almost no susceptibility to the NNRTI [21]. To note, PERV recombinant RT had a reduced susceptibility to all three NRTI compared with the RT of HIV-1, confirming the results obtained in cell cultures.

In the case of HIV-1, a resistance usually develops after treatment due to specific mutations in the reverse transcriptase [40,41]. In the case of PERV, no studies on resistances to antiretroviral drugs have been performed.

## 4. Inhibitors of Other PERV Enzymes

Inhibitors of the integrase were shown to be very effective in inhibiting PERV replication [24,25]. The viral enzyme integrase has a key role in the stable integration of the viral DNA copy into the cellular genome. Raltegravir and dolutegravir were shown to inhibit PERV effectively [24,25] (Table 1). When the catalytic domains of the integrase of PERV and HIV-1 were compared, complete conservation was observed [25], most likely accounting for the similar patterns of susceptibility to raltegravir and dolutegravir. In comparison with AZT, the integrase inhibitors raltegravir and dolutegravir were the most potent inhibitors of PERV.

Inhibitors developed for the protease of HIV-1 such as indinavir, nelfinavir, saquinavir, ritonavir, and amprenavir did not inhibit PERV [20] (Table 1), which may be explained by structural differences and the low sequence homology.

## 5. Monotherapy versus Combination Treatment

In using monotherapies for the treatment of HIV-1 infection, it quickly became clear that mutations were selected which are associated with resistance. This forced the development of cART. According to the World Health Organization (WHO) guidelines, the first-line antiretroviral therapy to treat HIV-1 infection for adults should consist of two NRTIs plus a NNRTI or an integrase inhibitor; e.g., tenofovir + 3TC (lamivudine or emtricitabine) + efavirenz [42]. After failure on a tenofovir + 3TC (lamivudine or emtricitabine)-based first-line regimen, AZT + 3TC as the NRTI backbone should be used in second-line regimens; after failure on an AZT or d4T (stavudin) + 3TC (lamivudine)-based first-line regimen, a second-line regimen of tenofovir + 3TC as the NRTI backbone should be used. Meanwhile, fixed dose combinations (i.e., multiple antiretroviral drugs combined into a single pill) were developed—e.g., truvada (tenofovir disoproxil fumarate + emtricitabine) and descovy (emtricitabine + tenofovir alafenamide).

At present, NRTI, NNRTI, and integrase inhibitors have been shown to inhibit PERV in culture (Figure 1, Table 1). It remains unclear whether these in vitro assays have translational value to the in vivo situation. In addition, no studies on resistance development in vitro have been performed. However, based on the use of these drugs for the treatment of HIV-1, dose levels, the bioavailability, the absorption, distribution, metabolism, and excretion (ADME), and the adverse side effects of these drugs are well known. It also remains unclear whether combinations of drugs that have been successful in the treatment of HIV-1 infections will be effective against PERV infections. It is clear from the facts summarized in the Introduction section that antiretroviral drugs are only needed in cases where patient monitoring indicates PERV transmission. Expecting new achievements in genetically modifying pigs (PERV-specific siRNA and CRISPR/Cas-based genome editing), the further development of effective drugs and combinations of drugs for PERV actually seems to be unnecessary. In the unlikely case that strategies based on siRNA and genome editing are not successful, meetings between virologists and transplant physicians should be held to discuss recommendations and agree on a consensus for potential treatment protocols. To speculate on the use of antiretroviral drugs as a pre-exposure prophylaxis (PrEP)—similar to that now often used to prevent HIV infection—would at this time be without a scientific basis.

## 6. Inhibitors of PERV Expression

Whereas the NRTI, NtRTI, NNRTI, and integrase inhibitors can be used as a pre-exposure treatment of potential transplant recipients (or in the worst case, to treat PERV infection), another strategy intends to reduce the expression of PERV in the donor pigs using drugs. Lower expression of mRNA implies lower production of viral proteins and infectious particles and a lower probability of infection. This strategy is comparable to the generation of pigs expressing PERV-specific siRNA in order to reduce PERV expression [26,27,28,29]. In one study, four inhibitors of HIV-1 gene expression were analyzed for their activity to inhibit PERV expression [23]. The fluoroquinolone derivative K-37 [43] and the bacterial product EM2487, produced from a *Streptomyces* species [44], were found to be potent and selective inhibitors of PERV expression (reducing the expression of viral mRNA) [23]. K-37 and EM2487 are also effective inhibitors of HIV-1 [39,40] and human T cell leukemia virus-1 (HTLV-1) [45,46] expression. The mechanism of action of both compounds is still unclear; it is assumed that K-37 interacts with a cellular factor.

## 7. Conclusions

Antiretroviral drugs such as inhibitors of the viral RT (mainly AZT) and integrase inhibitors have been shown to effectively inhibit PERV infections in vitro. Integrase inhibitors were the most potent drugs. Although there are still no investigations on resistance development after treatment, these drugs are available for combination antiretroviral therapies shown to be very successful in the case of HIV-1 treatment. The answer to the question of whether effective drugs (or combinations of drugs) to combat PERV infection should be developed, and whether this is actually needed, depends on the success of attempts to decrease PERV expression by RNA interference [26,27,28,29] or to inactivate all PERV proviruses in the pig genome by genome editing (e.g., CRISPR/Cas) [31]. If it becomes possible to generate pigs that cannot release infectious PERV, then of course no antiretroviral drugs will be required. However, it would still be useful to know whether such drugs are potent and available.

## Figures and Tables

**Table 1:** Inhibitory activity of clinically used human immunodeficiency virus-1 (HIV-1) inhibitors on porcine endogenous retrovirus (PERV).

Type of Inhibitor	Name	Inhibition of PERV	Reference
Nucleoside reverse transcriptase inhibitors (NRTIs)	AZT (azidothymidine, also called ZDV, zidovudin)	yes	[18,19,20,21,22,25]
	3TC (lamivudine)	no	[18,20]
	d4T (stavudin)	no/yes	[18,20,22]
Nucleotide reverse transcriptase inhibitors (NtRTIs)	TAV (adefovir)	yes	[22,25]
	TDF (tenofovir)	yes	[22,25]
Non-nucleoside reverse transcriptase inhibitors (NNRTIs)	EFV (efavirenz)	n.t.	[20,25]
	ETV (etravirin)	n.t.	[20,25]
	NVP (nevirapin)	no	[20,25]
Integrase inhibitors	RAL (raltegravir)	yes	[24,25]
	DTG (dolutegravir)	yes	[24,25]
Protease inhibitors	IDV (indinavir)	no	[18,20]
	NLV (nelfinavir)	no	[20]
	SQV (saquinavir)	no	[20]
	RTV (ritonavir)	no	[20]
	APV (amprenavir)	no	[20]

n.t., not tested.
